# EPC-EXs improve astrocyte survival and oxidative stress through different uptaking pathways in diabetic hypoxia condition

**DOI:** 10.1186/s13287-022-02766-7

**Published:** 2022-03-03

**Authors:** Manasi Suchit Halurkar, Jinju Wang, Shuzhen Chen, Ji Chen Bihl

**Affiliations:** 1 Department of Biomedical Sciences, Joan C Edwards School of Medicine, Marshall University, Huntington, WV 25755 USA; 2 Department of Pharmacology and Toxicology, Boonshoft School of Medicine, Wright State University, Dayton, OH 45435 USA

**Keywords:** Endothelial progenitor cells, Exosomes, Hypoxia, High glucose, microRNAs, Astrocytes

## Abstract

**Background:**

Hyperglycemia contributes to cardiovascular complications in patients with type 2 diabetes. We confirmed that high glucose (HG) induces endothelial dysfunction and cerebral ischemic injury is enlarged in diabetic mice. Stem cell-released exosomes have been shown to protect the brain from ischemic stroke. We have previously shown that endothelial progenitor cells (EPCs)-released exosomes (EPC-EXs) can protect endothelial cells from hypoxia/reoxygenation (H/R) and HG-induced injury. Here, we aim to investigate the effects of EPC-EXs on astrocytes under H/R and HG-induced injury and whether miR-126 enriched EPC-EXs (miR126-EPC-EXs) have enhanced efficacy.

**Methods:**

EPC-EX uptake and co-localization were measured by fluorescent microscopy using PKH26 and DAPI staining. miR-126 enrichment was achieved by transfecting with miR-126 mimics and quantified with real-time PCR. After co-incubation, cell death or injury was measured by using LDH (Lactate Dehydrogenase) assay. Oxidative stress/ROS (reactive oxygen species) generation was measured by DHE (Dihydroethidium) staining and lipid peroxidation assay.

**Results:**

The EPC-EXs were effectively taken up by the astrocytes in a concentration as well as time-dependent manners and were co-localized within the nucleus as well as the cytoplasm. Pathway uptake inhibitors revealed that the EPC-EXs are effectively taken up by the clathrin-mediated, caveolin-dependent, and micropinocytosis via PI3K/Akt pathway. H/R and HG-induced a cell injury which could be protected by EPC-EXs evidenced by decreased cell cytotoxicity, oxidative stress, and lipid peroxidation. Moreover, miR-126 overexpression could increase the level of miR-126 in astrocytes and enhance the protective effects of EPC-EXs.

**Conclusions:**

These results collectively indicate that the EPC-EXs could protect astrocytes against the HG plus H/R-induced damage.

## Introduction

Diabetes mellitus is a group of chronic diseases characterized by hyperglycemia. Over the last several decades, the global incidence and prevalence of diabetes mellitus have increased significantly. In the United States, the incidence of type 2 diabetes (T2D) has increased by 40% during the past decades. Diabetes mellitus is not merely a disorder of carbohydrate metabolism, but a cause of vascular disease affecting nearly all blood vessels. Indeed, cerebral vascular complication, such as ischemic stroke, is one of the most serious manifestations responsible for most of the morbidity, hospitalizations, and death that occur in patients with diabetes mellitus. Our previous studies have demonstrated that cerebral ischemia injury is enlarged, and the neurological recovery is delayed and held back in diabetic mice [1, 2]. With limited available treatments, researching the underlying mechanism and searching for novel strategies capable of protecting the brain from ischemic injury in diabetes are urgently needed.

Endothelial progenitor cells (EPCs) are referred to as a stem cell population which could be further differentiated into endothelial cells (ECs), thus known as the precursors of ECs [3]. EPCs have the properties of embryonal angioblasts leading to neovascularization and re-reendothelialization. Apart from these functions, EPCs are involved in wound healing, angiogenesis, tissue regeneration as well as remodeling [4, 5]. Previous studies involving EPCs have suggested that EPCs play a major therapeutic role in ischemic stroke [5, 6]. The underlying mechanism could be attributed to EPC-released exosomes (EXs) [7, 8]. EXs are extracellular particles, 30–100 nm, released from various cells and found to contain the essential biomolecules such as the mRNAs, microRNAs (miRs), proteins and lipids [9, 10]. Studies have suggested that EXs have the unique function of cellular signaling, wherein they carry information in the form of biomolecules from the parent cell to the recipient cell [10]. Along with this, they are involved in the facilitation of immune response, angiogenesis, wound healing, inflammation, and coagulation [11, 12]. They are also considered to be biomarkers in the diagnosis and prognosis of various medical conditions and have proved to have immense diagnostic potential over the years [13, 14]. Various studies in recent years have focused on the therapeutic efficacy of EPC-EXs. Li X. et al. and Zhang J. et al. proved that EPC-EXs accelerate cutaneous wound healing by promoting endothelial function and promoting angiogenesis via Erk1/2 signaling respectively [15, 16]. Our previous studies showed that EPC-EXs could protect endothelial cells from hypoxia/reoxygenation (H/R)-induced injury [8, 17]. Jia Y. et al. in 2019 suggested that EPC-EXs are involved in accelerating bone regeneration by regulating angiogenesis, in the distraction osteogenesis phase [18]. However, there are no published studies showing the effects of EPC-EXs on astrocytes in diabetes situations.

Among the molecules that EXs carry, miRs show the most important role in regulating cellular function. MicroRNA-126 (miR-126) is an endothelial-specific miR found to be expressed in a broad range of tissues, specifically the vascular system and highly vascularized tissues such as lungs and heart [19]. Like all other miRs, miR-126 also has specific functions in angiogenesis and thus is a prospective target for the regulation of vascular integrity and treating various vascular disorders [20, 21]. Studies have proved that priming of miR-126 in EPCs has been beneficial in enhancing their therapeutic efficacy in ischemic cerebral impairment [22, 23]. Our previous study has shown that miR-126 level is correlated with the function of EPC-EXs in diabetes [7]. We also showed that the miR-126 level is decreased in EPC-EXs in diabetes [7]. Therefore, our research questions are if overexpression of miR-126 could enhance the protective effects of EPC-EXs on high glucose (HG) and H/R-injured astrocytes?

## Methods

### Cell culture

Human EPCs (Celprogen, Torrance, CA) and human astrocytes (ASCs, ATCC, MD, USA) cell lines were used for this study. EPCs were cultured in EPC complete growth medium with serum and antibiotics in an incubator with 5% CO2 at 37 °C as previously published [24]. ASCs were cultured in ASC medium (ScienCell, Carlsbad, CA) with 10% fetal bovine serum (FBS), 5% ASC growth factor, and 5% penicillin/streptomycin (P/S) solution in a standard cell culture incubator. Culture medium was replaced every 2 days.

### Generation of miR-126 overexpressing EPC-EXs

The miR-126 mimic was used to generate miR-126 overexpressing EPC-EXs (miR126-EPC-EXs) per our previous publication [23]. Briefly, the EPCs were transfected with miR-126 mimics (1 nmol/L, Applied Biosystems) using Dharmafect 1 transfection reagent (Dharmacon) for 48 h in EPC complete growth medium. The complete medium was replaced with a serum-free medium for 48 h to promote the release of EXs. The culture medium was then collected for EXs isolation.

### EPC-EX isolation

EPCs were cultured in a serum-free medium for 48 h. After serum starvation, the culture medium was collected and centrifuged at 300×*g* for 6 min, followed by 2000×*g* for 20 min to remove the cells along with cell debris. The obtained supernatant was centrifuged at 20,000×*g* for 70 min, this allowed the pelleting of microvesicles (MVs). The obtained supernatant was subjected to ultracentrifugation at 170,000×*g* for 90 min to pellet the EXs. After ultracentrifugation, the supernatant was discarded, and the pellet was resuspended in 100 μl sterile-filtered phosphate buffer saline (PBS) for nanosight tracking analysis (NTA) or culture medium for co-incubation study.

### NTA analysis

The size and concentration of EPC-EXs were determined by NS300 (Nanosight, Amesbury, UK) [25]. NTA visualizes and measures particle size and concentration by utilizing light scattering and Brownian motion properties. It can detect the size distribution of particles in solution from 10 nm to 2 μm in diameter. The optimum particle concentration detected by NTA is ~ 10^7^–10^9^ particles/ml. For better detection, the EX samples were diluted with sterile-filtered phosphate-buffered saline (PBS) to a concentration of 10^7^–10^8^ particles/ml. After diluting the sample, 700 μl of the same was loaded in the instrument for movement tracking at the rate of 30 frames/s. The videos with particle movement were recorded at least 3 times per sample at different positions which were analyzed by the NTA software (version 3.5, Nanosight). The NTA results were produced as a mean of the 3 tests performed per sample and the particle concentration was calculated after considering the dilution factor.

### EPC-EX labeling

To label the EPC-EXs, PKH26 or PKH67, lipophilic-membrane dyes exhibiting red or green fluorescence were used per previous publications [8, 23]. The isolated EXs were incubated with 2 μl of PKH26 or PKH67 (2 × 10^−6^ M, Sigma-Aldrich) in 1 ml PBS for 5 min. To stop the reaction, 1 ml 1% BSA was added and incubated for one minute. The suspension was then ultracentrifuged at 170,000×*g* for 90 min to obtain the fluorescent-labeled EXs. The supernatant was discarded, and the pellet was resuspended in ASC medium for further co-incubation with the ASCs.

### Co-incubation of EPC-EXs with ASCs

ASCs were subjected to HG plus H/R to induce injury as an in vitro model of ischemic stroke in diabetes. For inducing HG + H/R injury, ASCs (80% confluent) were cultured in complete medium with 25 mM glucose for 24 h, and then placed in a hypoxia chamber (1% O_2_, 5% CO_2_, and 94% N_2_, Biospherixhypoxia chamber, NY, USA) for 6 h [2, 8]. Following this, the ASCs were reoxygenated for 24 h in a standard incubator (37 °C and 5% CO_2_), during which the EPC-EXs or miR126-EPC-EXs (3 × 10^9^ EX particles/ml) were added for co-incubation. The EPC-EXs or miR126-EPC-EXs were resuspended in complete ASC medium and then co-incubated with the ASCs for 24 h. Following this, the incorporation rate was measured by analyzing the fluorescent intensity of EPC-EXs (labeling with PKH) within the cells. Various assays were also performed to determine the function of EPC-EXs.

### EPC-EX uptake mechanism

To determine the EPC-EX uptake mechanisms by ASCs, we focused on the endocytic uptake pathways, including macropinocytosis, clathrin and caveolin-dependent pathways. For this, the cells were pre-treated for 30 min with various inhibitors: 80 μM dynasore (dynamin inhibitor, Sigma-Aldrich), 5 μM LY290042 (macropinocytosis inhibitor, Enzo), 10 μM pitstop 2 (clathrin inhibitor, Abcam) or 200 μM genistein (caveolin inhibitor, EMD Millipore). The concentration of the drugs was determined by following our previous studies [24]. After treatment, the cells were washed twice with PBS and then the PKH-labelled EPC-EXs were co-incubated with the ASCs for 24 h. Fluorescent images were obtained by a fluorescence microscope (EVOS, NY) and fluorescent intensity was quantified by flow cytometry analysis (Accuri C6 flow cytometer).

### PCR analysis of the miR-126 level

The levels of miR-126 in the EPCs, EPC-EXs and astrocytes were determined by Real-time PCR as we previously published [2, 23]. Briefly, the miRNA was extracted from the EPCs and EPC-EXs by using the TRIzol reagent and the RNA concentration was measured using NanoDrop 2000 Spectrophotometer (Thermo Fisher Scientific). cDNA was synthesized using PrimeScript RT reagent kit (Takara Bio Inc.) following the manufacturer’s instructions. qRT-PCR was carried out using miR-126 specific primers and SYBR Premix Ex Taq kit (Takara Bio Inc.) on a real-time PCR instrument (Bio-Rad). RNA U6 was used as an internal control. The RT primer: 5′-GTC GTA TCC AGT GCA GGG TCC GAG GTA TTC GCA CTG GATACG AC CGC ATT-3′. The primers of miR-126: 5′-AGG CGC TCG TAC CGT GAG TAA TA-3′ (forward); 5′-CCA GTG CAG GGT CCG AGG TA-3′ (reverse). The expression of U6 was used as an endogenous control for each sample. The primers of U6: 5′-CTCGCTTCGGCAGCACA-3′ (forward); 5′-AACGCTTCACGAATTTGCGT-3′ (reverse). The expression of miR-126 was normalized to U6 and calculated using 2 − ΔΔCT method.

### Intracellular reactive oxygen species (ROS) generation assay

Intracellular ROS generation, an effective method for determining oxidative stress in the cells was determined using dihydroethidium (DHE, Sigma-Aldrich) staining per our previous publications [24, 26]. DHE is a superoxide indicator which when oxidized primarily by superoxide resulting in 2-hydroxyethidium. The ASCs were cultured in a 6-well plate and incubated with 10 μM DHE solution (in dark) for 30 min at 37 °C. The cells were then observed under a fluorescence microscope and the percentage of DHE-positive cells was analyzed using the flow cytometer (Accuri C6).

### Cell cytotoxicity assay

Cellular cytotoxicity determination is carried out using LDH (lactate dehydrogenase, Fisher Scientific) cytotoxicity kit. ASCs were cultured in 96-well plates (in triplicate wells). After different treatments, ASCs were used for testing LDH activity. Absorbance was measured at 490 nm and 680 nm and calculations for the determination of percent cytotoxicity were carried out as per the manufacturer’s instructions.

### Lipid peroxidation assay

Lipid peroxidation refers to the degradation of cellular lipids due to the generation of reactive oxygen species within the cell. For the determination of lipid peroxidation, a sensitive fluorescent reporter BODIPY 581/591 C11 reagent (BODIPY dye, Invitrogen) was used. The phenylbutadiene portion of the dye allows the shift of fluorescence emission peak from 590 nm-red to 510 nm-green upon oxidation. To determine the lipid peroxidation, after different treatments, ASCs cultured in a 6-well plate were incubated with 10 μM BODIPY reagent for 30 min at 37 °C. The ratio of reduction (590 nm)/oxidation (510 nm) was derived by reading the fluorescence intensities at the separate wavelengths on Cytation 5 plate reader (Biotek) while the percent lipid peroxidation was analyzed by the flow cytometer.

### Statistical analysis

All data are presented as mean ± SD. Two group comparison was analyzed by Student’s t-test. Multiple comparisons were analyzed by two-way ANOVA (SPSS version 16.0; SPSS, Chicago, IL, USA) followed by the Tukey test. For all tests, a *p* value < 0.05 was considered significant.

## Results

### EPC-EXs were uptaken by ASCs in concentration and time-dependent manners

**Fig. 1:**
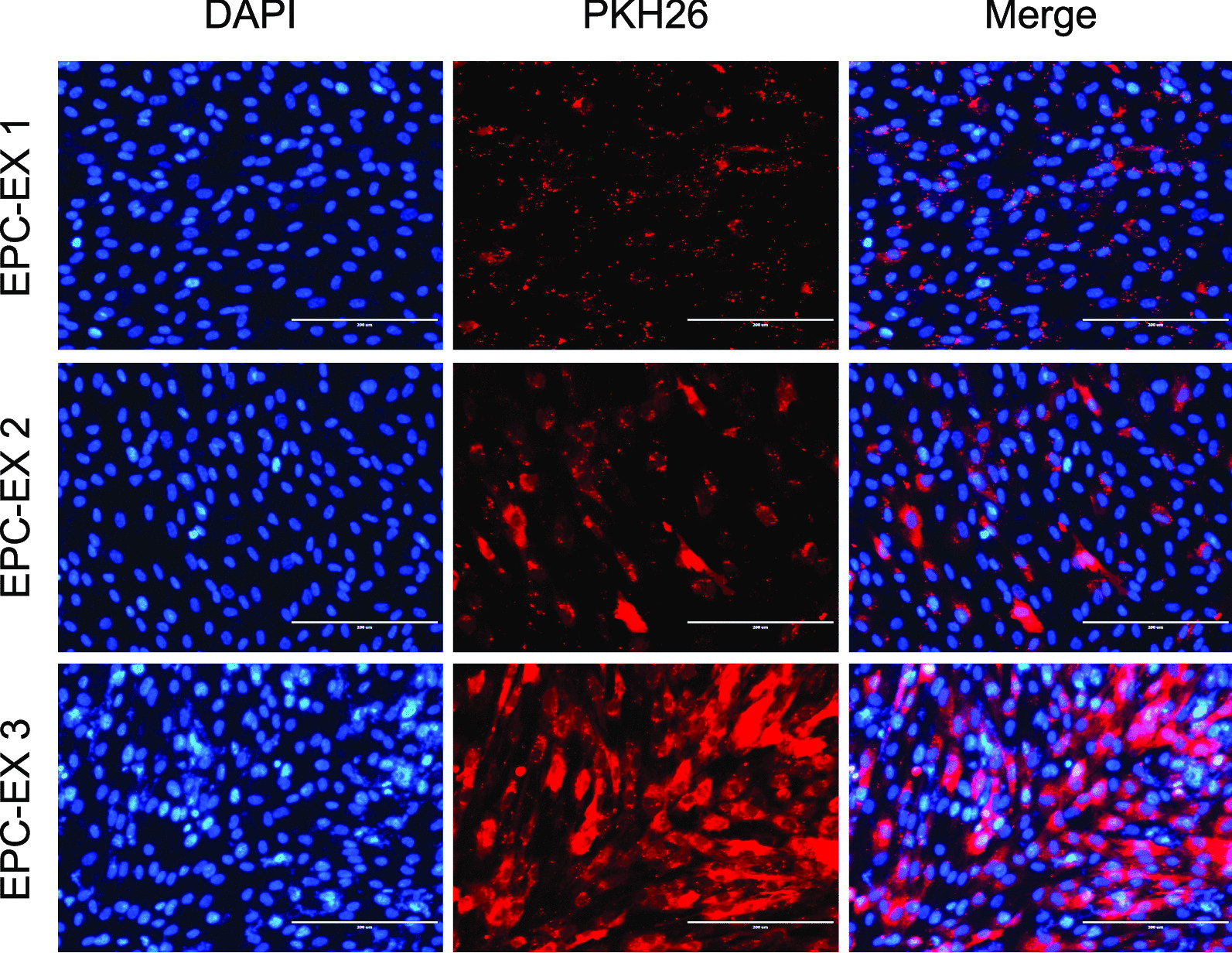
Concentration-dependent uptake of EPC-EXs by ASCs. Representative images of PKH26 stained EPC-EXs merged with ASCs, observed under the fluorescence microscope after 24 h co-incubation. Red: PKH-labeled EPC-EXs; Blue: nucleus of ASCs. Scale bars: 200 μm. EPC-EX 1: 1 × 10^9^ particles/ml; EPC-EX 2: 2 × 10^9^ particles/ml; EPC-EX 3: 3 × 10^9^ particles/ml. Data represents mean ± SD, *n* = 3/group

**Fig. 2:**
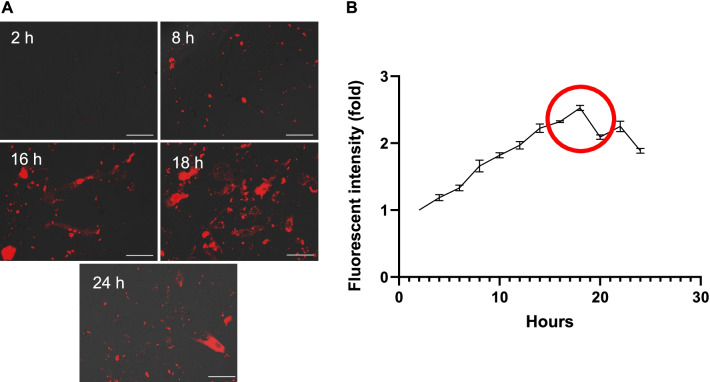
Time-dependent uptake of EPC-EXs by ASCs. **a** Representative images of PKH26 stained EPC-EXs merged with ASCs at various time points. Red: PKH-labeled EPC-EXs. Scale bars: 200 μm. **b** Data summary representing fold change fluorescence for time-based uptake of PKH26 stained EPC-EXs by ASCs over 24 h period. Fluorescence images were obtained every 2 h along with flow cytometry data. Data represent mean ± SD, *n* = 6/group

For the concentration-based study, cultured ASCs were co-incubated with PKH26 labelled EPC-EXs at 3 different concentrations-EX1: 1 × 10^9^ particles/ml, EX2: 2 × 10^9^ particles/ml and EX3: 3 × 10^9^ particles/ml for 24 h. The fluorescence images were recorded after 24 h indicating the labeled EX particles merged with the ASCs. The intensity of the exhibited red fluorescence suggesting that the uptaking ability of the EXs by the ASCs. The images suggested that the EXs were uptaken by the cells in a concentration-based pattern, wherein the fluorescence exhibited increased as the concentration of EXs increased (Fig. 1). Therefore, for the time-dependent study, PKH26 labeled EXs were co-incubated with the ASCs at a concentration of 3 × 10^9^ particles/ml for 24 h. The fluorescent images were recorded every 2 h. Again, the fluorescence intensity proved to be the basis for the uptaking of these particles, merged with the cells. It was observed that the uptake was gradual as time passed, greater uptake and fluorescence intensity was observed at 16–18 h. This fluorescence gradually decreased until 24 h suggesting the EXs being consumed by the cells, hence the loss in fluorescence (Fig. 2a). Fluorescence fold change was recorded by flow cytometry at every time point (Fig. 2b).

### EPC-EXs were uptaken by ASCs via macropinocytosis, caveolin-dependent, and clathrin-mediated pathways

**Fig. 3:**
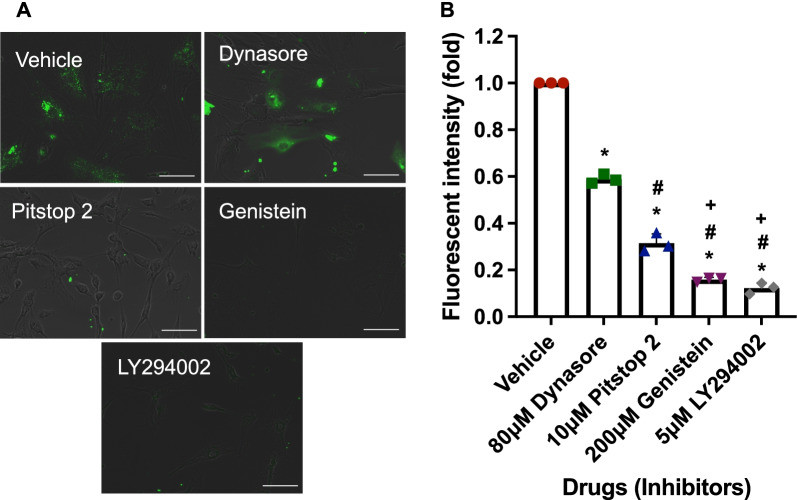
Mechanisms of uptake for EPC-EXs by ASCs. **a** Representative fluorescent images of PKH67 labeled EPC-EXs merged with ASCs after 30 min treatment by vehicle, 80 μM Dynasore, 10 μM Pitstop 2, 200 μM Genistein and 5 μM LY294002, over an incubation period of 24 h. **b** Fluorescence intensity fold change levels determined by flow cytometric analysis. Data represent mean ± SD, *n* = 3/group. *p < 0.05 versus Vehicle, ^#^*p* < 0.05 versus Dynasore, ^+^*p* < 0.05 versus Pitstop 2

To determine the uptake mechanisms, ASCs were treated with various synthetic drugs which allowed the inhibition of the uptake of PKH67 labeled EPC-EXs (green). These drugs were dynamin, macropinocytosis, clathrin, and caveolin inhibitors (respectively: Dynasore, LY294002, Pitstop 2, and Genistein). It was observed that the EX uptake significantly decreased after the treatment of these inhibitors in comparison to the no-treatment group (vehicle, Fig. 3a). The green fluorescence intensity suggested the uptake and merging of the EXs with the cells. Decreased fluorescence suggested decreased uptake of the labeled EXs by the cells hence, the inhibition of uptake had occurred after the drug activity. Fluorescence fold change was determined by flow cytometry (Fig. 3b). Summarized data showed that the incorporation rate of ASCs with EPC-EXs was significantly lower in the inhibitor group when compared to the vehicle group (*p* < 0.05, vs*.* vehicle). This is consistent with the fluorescent intensity shown in the representative images. Among the different pathway inhibitors, Dynasore had the least effects on inhibiting the uptaking. Pitstop 2 had modest effects, while LY294002 and Genistein had the most inhibitory effects. These suggest that clathrin and caveolin might be the main uptaking pathways for EPC-EXs in ASCs.

### The enhanced protective effect of miR126-EPC-EXs H/R-induced cytotoxicity of ASCs

**Fig. 4:**
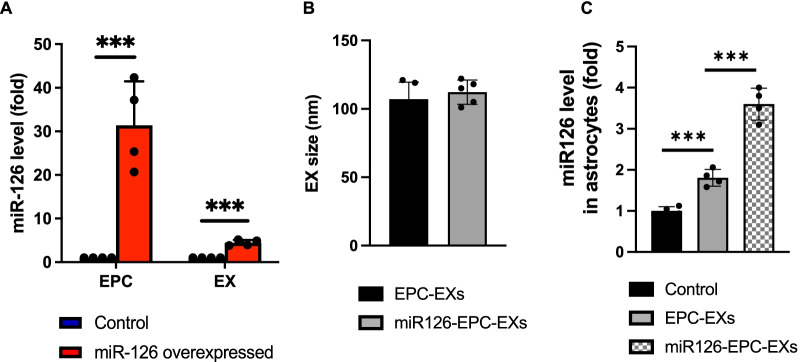
The effects of miR-126 mimics transfection on the levels of miR-126 in EPCs, EPC-EXs and astrocytes. **a** Summarized data represents the fold change in levels of miR-126 expression in miR-126 transfected EPCs and their released EXs. **b** The size and concentration of EPC-EXs in different groups. **c** The level of miR-126 in astrocytes after co-incubation in different groups. Data are represented as mean ± SD, *n* = 4–6/group, ***p < 0.05 versus control

To obtain miR-126 overexpressing EPC-EXs, EPCs were transfected with miR-126 mimics and EXs in the culture medium were isolated. The level of miR-126 in both EPCs and EPC-EXs was analyzed by qRT-PCR. Results (Fig. 4a) showed that the level of miR-126 in transfected EPCs was 30 folds of control (*p* < 0.05, vs*.* control), while that for the isolated EXs was found to be around 5 folds (*p* < 0.05, vs*.* control). This data confirmed the successful generation of miR126-EPC-EXs. In addition, the NTA data showed that miR-126 transfection did not affect the size and concentration in different groups (Fig. 4b). More importantly, the level of miR-126 in the astrocyte was determined after co-incubation. The PCR data showed that the miR-126 level of astrocytes was slightly increased in the EPC-EXs group, but significantly increased in the miR126-EPC-EXs group after co-incubation (Fig. 4c). This data suggests the successful transferring of miR-126 to the astrocytes by exosomes.

**Fig. 5:**
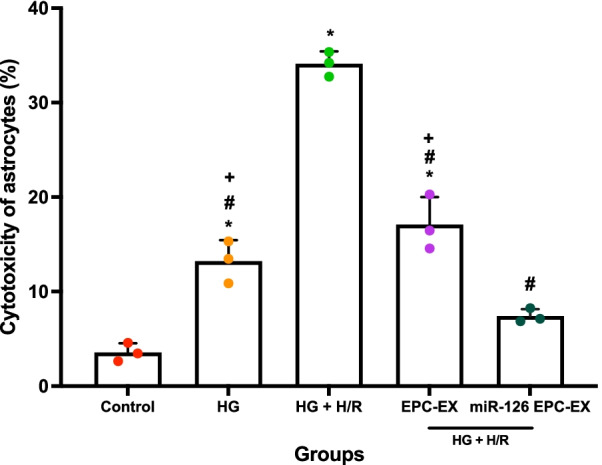
Effect of miR-126-EPC-EXs on cell cytotoxicity after HG + H/R-induced ASCs injury. Summarized colorimetric absorbance readout data calculated to represent percent cytotoxicity. Control: no injury or treatment, HG: High glucose, HG + H/R: High glucose + hypoxia/reoxygenation, EPC-EX: Endothelial progenitor cells-derived exosomes, miR126-EPC-EX: microRNA-126 overexpressing EPC-EXs. Data are represented as mean ± SD, *n* = 3/group. *p < 0.05 versus Control, ^#^*p* < 0.05 versus HG + H/R, ^+^*p* < 0.05, versus miR126-EPC-EX

To determine the effect of miR-126 overexpression in EPC-EXs on cellular cytotoxicity, LDH assays were performed. The data showed that HG-induced significant cytotoxicity of ASCs (Fig. 5, *p* < 0.05, vs*.* control), while HG + H/R further increased cytotoxicity (*p* < 0.05, vs*.* HG). This confirmed the injury model of ASCs by HG + H/R. As we expected, EPC-EXs could decrease the cytotoxicity of ASCs when compared to HG + H/R groups (*p* < 0.05). More interestingly, miR-126 overexpression further decreased the cytotoxicity of ASCs (Fig. 5, *p* < 0.05, vs*.* HG + H/R and EPC-EXs), suggesting the enhanced effect of miR126-EPC-EXs on the injured cells.

### The protective effect of miR126-EPC-EXs on H/R-induced ROS in ASCs

**Fig. 6:**
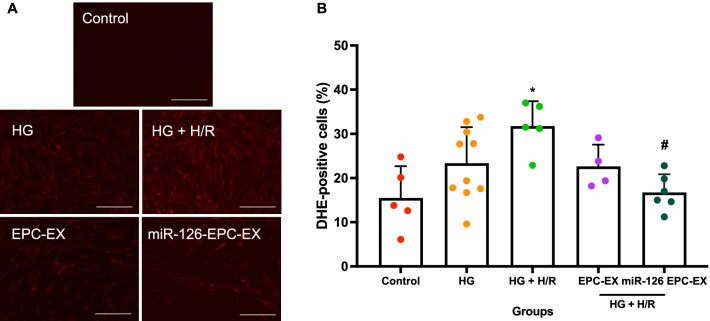
Effect of miR-126-EPC-EXs on ROS production after HG + H/R-induced ASCs injury. **a** Representative DHE staining fluorescent images exhibiting bright red fluorescence. Scale bars: 200 μm. **b** Summarized flow cytometry analysis representing percent DHE-positive cells. Control: no injury or treatment, HG: High glucose, HG + H/R: High glucose + hypoxia/reoxygenation, EPC-EX: Endothelial progenitor cells-derived exosomes, miR126-EPC-EX: microRNA-126 overexpressing EPC-EXs. Data are represented as mean ± SD, *n* = 4–10/group. *p < 0.05 versus Control, ^#^*p* < 0.05 versus HG + H/R

As discussed previously, ROS generation is a key factor in cell damage and death. Here, we determined if miR126-EPC-EXs influence ROS generation, thus affecting oxidative stress and lipid peroxidation. DHE staining exhibited bright red fluorescence (Fig. 6a), detected by fluorescence microscope while the percent DHE-positive cells were determined by flow cytometry (Fig. 6b). The summarized data showed that HG + H/R could increase the level of ROS in ASCs (*p* < 0.05 vs*.* control, Fig. 6b). It was found that miR126-EPC-EXs decrease the ROS generation significantly (*p* < 0.05 vs*.* HG + H/R, Fig. 6b), however, there was no significant difference observed between the effects of EPC-EXs and miR126-EPC-EXs groups (*p* > 0.05).

### The protective effect of miR126-EPC-EXs on H/R-induced lipid peroxidation in ASCs

**Fig. 7:**
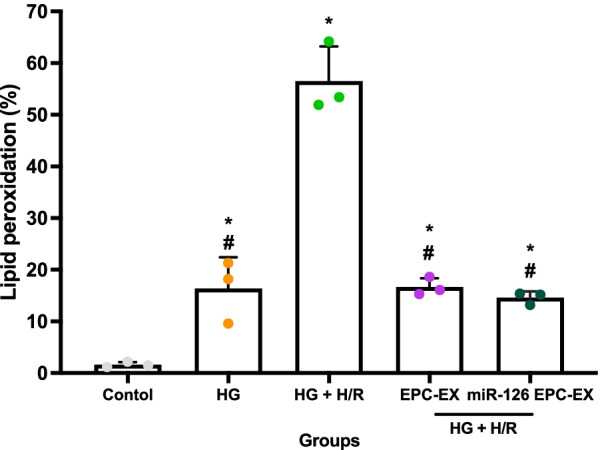
Effect of miR-126-EPC-EXs on lipid peroxidation after HG + H/R-induced ASCs injury. Summarized flow cytometry analysis representing percent intracellular lipid peroxidation. Control: no injury or treatment, HG: High glucose, HG + H/R: High glucose + hypoxia/reoxygenation, EPC-EX: Endothelial progenitor cells-derived exosomes, miR126-EPC-EX: microRNA-126 overexpressing EPC-EXs. Data are represented as mean ± SD, *n* = 3/group. *p < 0.05 versus Control, ^#^*p* < 0.05 versus HG + H/R

To determine the intracellular lipid peroxidation, the cells were stained with BODIPY dye and the ratio of reduction (590 nm)/oxidation (510 nm) was determined by flow cytometry analysis. Similar to the cytotoxicity results, HG-induced lipid peroxidation in ASCs (*p* < 0.05 vs*.* control, Fig. 7), while HG + H/R induced dramatically lipid peroxidation (*p* < 0.05 vs*.* control and HG, Fig. 7). EPC-EXs and miR126-EPC-EXs both significantly decreased the lipid peroxidation in ASCs (*p* < 0.05 vs*.* HG + H/R, Fig. 7). However, there was no significant difference observed between the effects of EPC-EXs and miR126-EPC-EXs groups (*p* > 0.05).

## Discussion

It has been widely accepted that diabetes is a risk factor for vascular diseases, such as stroke and atherosclerosis. In diabetic patients, ischemic cerebral damage is exaggerated, and the outcome is poor. Traditional stroke studies usually explore treatments by focusing on ischemic stroke itself; however, on the clinical side, the patients are often accompanied by different risk factors, such as aging, hypertension, diabetes, etc. To better mimic the patient’s situation, we considered both factors: diabetes and ischemic stroke in the current study. Therefore, the high glucose and H/R injury model was applied to astrocytes to mimic the diabetic ischemic stroke in an in vitro system.

We have used the high glucose and H/R injury model in cells, to investigate the protective effects of EPC-EXs against high glucose and H/R-indued injury. As we know, brain cells require a sufficient amount of oxygen and glucose to generate ATP and maintain membrane potential for normal functioning [27]. During ischemic stroke, as the artery that supplies blood to the brain is blocked, the brain cells do not receive blood supply properly and so they are glucose and oxygen-deprived. This results in ATP level depletion and rises the intracellular calcium levels [28], which causes mitochondrial injury, free radical generation, and damages the nucleus and, cell membrane, and eventually leading to cell death [28, 29]. As expected, our data showed that high glucose and H/R injury increased the cytotoxicity, oxidative stress and eventually reduced the viability of astrocytes, indicating the success of the injury model.

As we have previously shown that the infarct size of ischemic stroke is enlarged in diabetic mice which are associated with the reduced EPC number and function [1]. EPCs play an important role in maintaining endothelium hemostasis and integrity [3]. Therefore, enhancing EPC function or infusion of exogenous EPCs is a useful therapeutic avenue for ischemic stroke. As expected, we demonstrated that the administration of EPCs provides therapeutic effects on ischemic stroke [1, 5]. More recently, we found that this therapeutic effect of EPCs might be ascribed to their released exosomes [17, 23]. We have reported the protective effects of EPC-EXs on endothelial cells [8]. Here, we further investigated the protective effects of EPC-EXs on astrocytes under high glucose and H/R injury. First, we demonstrated that EPC-EXs could be uptaken by astrocytes after co-incubation by showing the incorporation of EPC-EXs within the cytoplasm and nucleus of astrocytes (Fig. 1). As the literature suggests, there are various pathways for EX uptake which predominantly are based on the cell line being studied. Copper et al. [30] reported that uptake of exosomes by adipose-derived stem cells is related to clathrin- and caveolin-dependent endocytosis. Li et al. [24] showed that dynasore (dynamin inhibitor) did not inhibit the uptake of EPC-EX by neurons, while LY290042 (macropinocytosis inhibitor), pitstop 2 (clathrin inhibitor), or genistein (caveolin inhibitor) significantly decreased the uptaking of exosomes by neurons. To determine the EPC-EX uptake by astrocytes, we focused on the endocytic uptake narrowing it down to macropinocytosis, clathrin-mediated and caveolin-dependent pathways. The inhibitors of macropinocytosis, clathrin-mediated endocytosis, and caveolin-dependent endocytosis pathways were used to determine the uptake mechanism of astrocytes. In this study, we isolated the EXs from EPCs that were uptaken by the ASCs via. Data showed that the uptake was concentration and time-dependent with the highest uptake rate observed at the concentration of 3 × 10^9^ particles/ml (Fig. 1) at 16–18 h (Fig. 2). We also observed a slight decrease of fluorescent intensity suggesting the consumption of EXs uptaken by the astrocytes. Moreover, we found that three pathways (macropinocytosis, clathrin, and caveolin) are all involved in the uptaking of EPC-EXs by astrocytes which were dominated by the micropinocytosis and caveolin-dependent endocytosis pathways (Fig. 3). These results suggest that EPC-EXs are effectively taken up by the astrocytes in concentration and time-dependent patterns by 3 different uptake mechanisms.

EXs are found to contain DNA, RNA, mRNA, miRNA, lipids, and proteins, which are responsible for cell-to-cell signaling transduction. This property of EXs exemplifies them as novel therapeutic agents also, qualifying them for being involved in drug delivery. miR-126 is a highly expressed endothelial-specific miRNA, located on the human chromosome 9. It has been noted that miR-126 has proved to be therapeutically efficacious in cerebral impairment due to ischemia [23], and the expression of miR-126 in diabetes is decreased [7]. We have shown that high glucose could decrease the expression of miR-126 in EPCs and EPC-EXs. While EPC-EXs provide beneficial effects on ischemia injury, and miR-126 could serve as the therapeutic target for diabetes, we hypothesize that the combination of EPC-EXs and miR-126 could provide enhanced protective effects on diabetic ischemic stroke. Hence, we overexpressed the levels of miR-126 in EPCs by transfecting EPCs with miR-126 mimics. The PCR results from this study showed a ~ 30-fold increase in miR-126 levels in the transfected EPCs when compared to the controls. More importantly, we found their released EXs also have increased miR-126 expression (Fig. 4). This indicates that the miR-126 is effectively transfected and overexpressed in the EPC-EXs which could, in turn, provide therapeutic efficacy. By using the NTA, we did not find any changes on the size and concentration of exosomes in different groups. Moreover, we confirmed that the miR-126 could be transferred to astrocytes by exosomes after co-incubation evidenced by the increased level of miR-126 in miR126-EPC-EXs group. Interestingly, we found that the miR-126 level of astrocyte in EPC-EXs group was also increased. This could be explained by their endogenous carried miR-126 as we previously reported and be the mechanism underlying the protective effects of EPC-EXs we observed.

Afterward, we performed co-incubation of EPC-EXs or miR126-EPC-EXs with the astrocytes and then measured the survival and oxidative stress of astrocytes to determine the function of miR126-EPC-EXs. The brain consists of neurons and a much higher number of glial cells. Astrocytes are a sub-type of glial cells in the central nervous system. They communicate with each other, by which they control brain functions. Though almost all studies have been performed from the viewpoint of neurons, recent studies have uncovered the active roles of astrocytes in modulating brain injury and repair during ischemic stroke. Here, we investigate the role of EPC-EXs and miR-126 in protecting astrocytes in an in vitro diabetic ischemic stroke model. We found that EPC-EXs could promote astrocytes survival by decreasing the injury of astrocytes (Fig. 5). More interestingly, overexpression of miR-126 enhanced these protective effects. Given oxidative stress plays an important role in early brain cell injury, we also detected ROS production and lipid peroxidation after different treatments. EPC-EXs significantly decreased the ROS generation represented by the bright red fluorescence and the DHE-positive cells. EPC-EXs were also found to decrease the ratio of reduction/oxidation of intracellular lipids and lipid peroxidation within the astrocytes injured by HG + H/R. These results demonstrate the enhanced therapeutic efficacy of miR126-EPC-EXs in attenuating the ROS generation by HG + H/R-induced astrocyte injury. Similarly, miR126-EPC-EXs significantly reduced cellular cytotoxicity and the ROS generation elucidated by the bright red DHE-stained cells as well as the summarized flow cytometry data. This data proclaims that miR-126 is responsible for the protective effects of EPC-EXs on HG and H/R-injured astrocytes by alleviating oxidative stress. The mechanisms could be through their downstream pathways. miR-126 directly targets vascular endothelial growth factor A (VEGF-A), vascular cell adhesion molecule 1 (VCAM-1), insulin receptor substrate 1 (IRS-1), sprouty related EVH1 domain containing 1 (SPRED-1) and PI3K (phosphoinositol-2-kinase) regulatory subunit p85 beta (PIK3R2) to regulate angiogenesis, cell survival and maintenance of the vascular structure. Hence, miR-126 plays a vital role in vascular hemostasis and cellular repair after ischemia injury. Further studies would be carried on determining the potential targets for miR-126 in the astrocyte and the related underlying mechanisms.

## Conclusions

Our data from this study shows that the EPC-EXs are efficiently uptaken by the astrocytes in a time and concentration-dependent manner. The major pathways for the mechanism of EX uptake in ASCs are macropinocytosis, clathrin-mediated endocytosis, and caveolin-dependent endocytosis. EPCs transfected with miR-126 mimics release miR-126 overexpressing EPC-EXs. The treatment of EPC-EXs resulted in reduced oxidative stress, lipid peroxidation, and cytotoxicity, which could be further enhanced by miR-126 overexpression. In summary, our study demonstrates that miR126-EPC-EXs provide enhanced protective effects on astrocytes under HG + H/R-induced injury.

## Data Availability

The datasets used and/or analyzed during the current study are available from the corresponding author on reasonable request.

## Abbreviations

EXs: Exosomes
EPCs: Endothelial progenitor cells
H/R: Hypoxia/reoxygenation
HG: High glucose
miR: MicroRNA
EPC-EXs: Endothelial progenitor cell-released exosomes
miR126-EPC-EXs: MiR126 overexpressed EPC-EXs
ASC: Astrocytes
ROS: Reactive oxygen species
DHE: Dihydroethidium
LDH: Lactate dehydrogenase
VEGF-A: Vascular endothelial growth factor A
VCAM-1: Vascular cell adhesion molecule 1
IRS-1: Insulin receptor substrate 1
SPRED-1: Sprouty related EVH1 domain containing 1
PIK3R2: PI3K (phosphoinositol-2-kinase) regulatory subunit p85 beta
